# Radiotherapy-induced Cherenkov luminescence imaging in a human body phantom

**DOI:** 10.1117/1.JBO.23.3.030504

**Published:** 2018-03-20

**Authors:** Syed Rakin Ahmed, Jeremy Mengyu Jia, Petr Bruza, Sergei Vinogradov, Shudong Jiang, David J. Gladstone, Lesley A. Jarvis, Brian W. Pogue

**Affiliations:** a Thayer School of Engineering at Dartmouth, Hanover, New Hampshire, United States; b University of Pennsylvania, Departments of Biophysics and Biochemistry and of Chemistry, Philadelphia, Pennsylvania, United States; c Geisel School of Medicine at Dartmouth, Department of Medicine, Hanover, New Hampshire, United States; d Dartmouth–Hitchcock Medical Center, Norris Cotton Cancer Center, Lebanon, New Hampshire, United States

**Keywords:** Cerenkov, radiation, therapy, linac, phosphorescence

## Abstract

Radiation therapy produces Cherenkov optical emission in tissue, and this light can be utilized to activate molecular probes. The feasibility of sensing luminescence from a tissue molecular oxygen sensor from within a human body phantom was examined using the geometry of the axillary lymph node region. Detection of regions down to 30-mm deep was feasible with submillimeter spatial resolution with the total quantity of the phosphorescent sensor PtG4 near 1 nanomole. Radiation sheet scanning in an epi-illumination geometry provided optimal coverage, and maximum intensity projection images provided illustration of the concept. This work provides the preliminary information needed to attempt this type of imaging *in vivo*.

## 1 Introduction

All optical imaging techniques suffer from diminished spatial resolution with increasing depth into tissue because of the severe light scattering present.^1^ Yet, in recent studies it has been shown that Cherenkov-based excitation of molecular probes is a feasible way to image deep into tissues, over several centimeters, with submillimeter resolution.^2^ One important application of this is luminescence-based sensing of oxygen over macroscopic depths into tissue. The available depth of sampling is appropriate for imaging oxygenation in lymph nodes. In this study, the feasibility of imaging oxygen in lymph node-sized objects in a human body phantom was assessed to estimate applicability of this luminescence lifetime sensing technology in human imaging.

Cherenkov radiation is produced in all tissues undergoing treatment with Megaelectron Volt photons or electrons,^3^ generating broadband optical radiation that can be used to excite molecular probes.^4^^,^^5^ Clinical linear accelerators (linacs) operate in pulsed mode with ∼4-μs bursts,^6^^,^^7^ allowing for excitation of phosphorescent probes that have 10- to 100-μs range emission decay times.^8^ Previous experiments demonstrated this luminescence imaging with Cherenkov excitation in basic tissue phantoms and in rodents,^9^^,^^10^ to image tissue oxygenation, with time-gated signals that were sensitive to the luminescence quenching by oxygen. The key advantage of the method was to image centimeters into tissue while retaining submillimeter spatial resolution. At the same time, imaging oxygen is relevant in radiotherapy since it is a radiosensitivity factor that correlates with outcome.^11^

In this work, sheet-shaped radiation beams from a linac were used to produce Cherenkov photons, to excite luminescence of probes in a volume largely confined to the irradiation sheets. Thus, by shaping the x-ray beam into a thin sheet, images of Cherenkov-excited luminescence from a planar slice within the tissue can be observed. Analogous to light sheet microscopy, a series of luminescence images can be taken at different depths, allowing for three-dimensional (3-D) volumetric rendering. This imaging approach has been termed Cherenkov-excited luminescence scanned imaging (CELSI).^2^^,^^10^ Knowing the position of the excitation plane allows for depth-variant attenuation correction, and postprocessed 3-D images show the luminescence distribution from within the tissue.

**Fig. 1:**
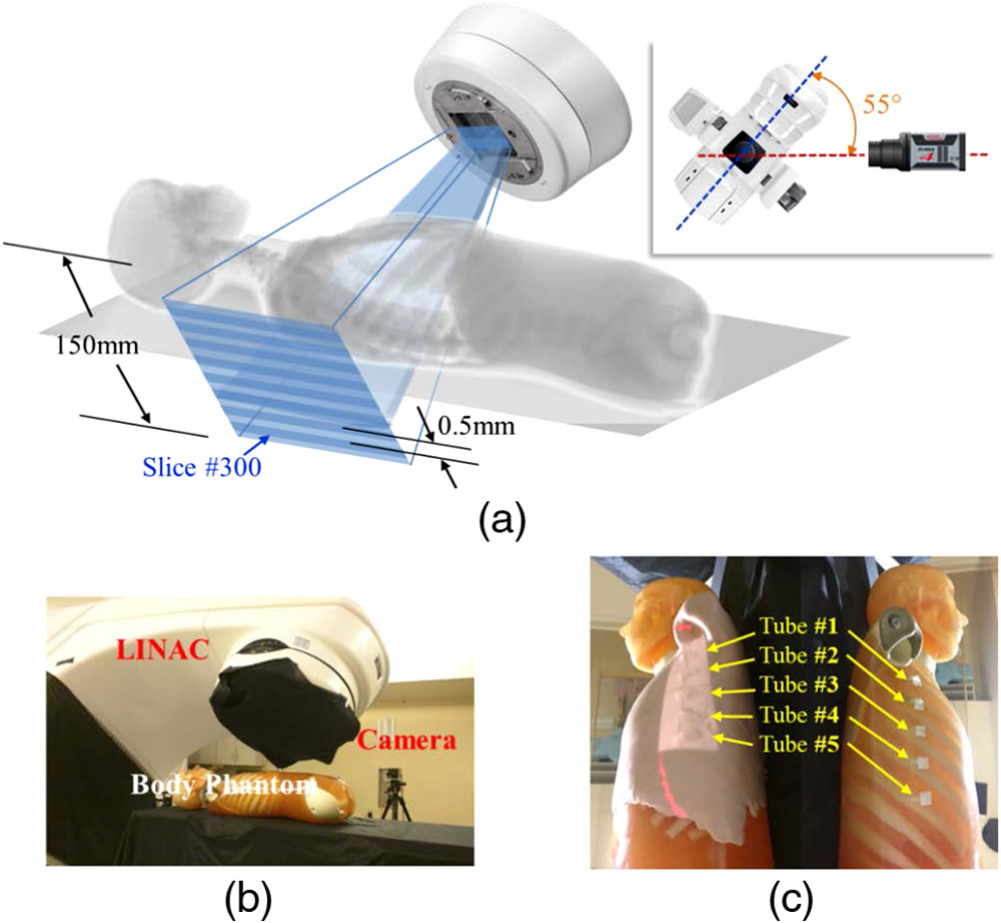
Measurement geometry for the body phantom is shown with (a) a schematic of the geometry, (b) a photograph of the setup, and (c) the body phantom with skin on (left) and without skin (right) as shown with five tubes fixed onto the lateral rib area.

The aim of this study was to study CELSI in a human body geometry, to demonstrate the capability of imaging local oxygenation in regions similar in size to lymph nodes, as shown in Fig. 1. Specifically, the effects of depth, concentration, and minimum radiation dose were examined here.

## 2 Materials and Methods

### 2.1 Phantoms and Geometry

Depth and concentration range evaluation experiments were carried out to assess the signal-to-noise ratio (SNR) and signal-to-background ratio for phosphorescent objects at varying depths. The phosphorescent probe used in our experiments was PtG4, which has a well-demonstrated oxygen-dependent luminescence lifetime, characterized in solution^12^ as well as in our previous animal study.^2^ Eppendorf tubes containing 0.5-mL solution of PtG4 of varying concentrations (1 to 100  μM) were positioned at different depths (0 to 3.5 cm) to assess imaging capability with concentration and depth. A semi-infinite tissue phantom, of 1% aqueous solution of intralipid and 1% bovine blood was used to mimic the optical properties of the human tissue.

A full torso PBU-50 phantom (Kyoto Kagaku America Inc., Torrance, California) was used with two sets of measurements. First, Eppendorf tubes were used with 50  μL of 25-μM solution of PtG4 (0.5 nanomoles) in phosphate buffered saline (PBS), and comparing with controls of just PBS and an empty tube. In the second set, five 0.2-mL Eppendorf tubes were used, each containing (1) 50  μL of solution of PtG4 at concentrations: 1, 5, 10, 20, and 25  μM, for total quantities of 0.05, 0.25, 0.5, 1.0, and 1.25 nanomoles, respectively. The tubes were placed in the lateral rib region of the phantom at a sufficient distance from one another to prevent spatial overlap of their signals.

In both experimental sets, the phantom was imaged both with and without “skin layer” that was mimicked using tissue colored modeling clay (SuperSculpey^®^ Original). Prior to the CELSI experiments, we validated the tissue-like optical properties of the used modeling clay by measuring its absorption and scattering coefficients.^13^ Using a custom tissue spectroscopy device, the DOSI system,^14^ we measured values across the spectral range of 650 to 1000 nm. The spectral range of PtG4 phosphorescence emission is peaked at 772 nm, the absorption and scattering coefficients of the clay ($\mu_{a}=0.014\;{\mathrm{mm}}^{-1}$, $\mu_{s}^{\prime }=0.55\;{\mathrm{mm}}^{-1}$ match reasonable values of skin and/or adipose human tissue. A clinical x-ray CT of the body phantom, with 2.5-mm slice thickness, was obtained to facilitate postprocessing image overlay (LightSpeedCT, GE Healthcare, Chicago, Illinois).

### 2.2 Linear Accelerator

In each experiment, a Varian linac (2100CD, Varian Medical Systems, Palo Alto, California) was used to deliver six MV x-ray photon beams to the phantoms in the form of thin sheets. Multileaf collimators (MLC) (Millennium 120MLC, Varian Medical Systems) were used to shape and vertically translate the beam profile adaptively. Each sheet was 200-mm wide and 5-mm thick. The MLC leaves translated a total distance of 50 mm with a 0.2-mm step—this resulted in a total of 250 x-ray sheet positions along the length of the treatment area. The maximum dosage of radiation delivered by the linac was 20 MU/position, with up to 25 overlapping positions (5 mm moved in steps of 0.2 mm); this resulted in a maximum of 20×25=500  MU being delivered to the volume as defined by a single beam sheet—roughly equivalent to a maximum absorbed dose of 5 Gy.

### 2.3 Luminescence Emission Imaging

Images were captured by a gated, intensified charge-coupled device (ICCD, PI-MAX4 1024i, Princeton Instruments) with a 135-mm lens (Nikon), using the associated LightField software. An epi-illumination configuration was used, with the linac gantry oriented at 145 deg and in the same side as the ICCD. The ICCD gate delays and widths were adjusted with 0.05 μs delay and 4 μs width for Cherenkov imaging, whereas a 4.2  μs delay and 70  μs width was used for phosphorescence imaging, and 1500  μs delay and 70  μs width used for background. Cherenkov, phosphorescence, and background images were acquired with 100× gain on the intensifier. The image intensifier was gated by a predefined number of pulses, whereas the CCD integrated the signal prior to readout. Different values of this approach to accumulations on the chip (AoC) were used for each of the Cherenkov, phosphorescence, and background images for all body phantom CELSI experiments. Room-light images were acquired with 1× gain and 1 AoC. An 8-MHz analog-to-digital conversion rate was used with 2×2  pixel hardware binning upon readout, resulting in 512×512  pixel images.

### 2.4 Image Processing

Image processing was carried out in MATLAB and Python. The protocol first involved background subtraction, followed by a depth stack (z-stack) median filter, across four frames in sequence, resulting in 250 frames. Next, each image was background corrected relative to its own residual background by subtracting the median from a background region of each image in the z-stack, followed by a 2-D (5×5) and a 3-D (5×5×5) spatial median filter. Finally, a 3-D Gaussian Filter with FWHM=4.7pixels was applied. Maximum-intensity projection (MIP) images were generated along the z-stack. The processed images were overlaid on the room-light image using both ImageJ and MATLAB Overlay GUI.^15^ Subsequently, videos were generated using ImageJ, showing the translation of the sheet beam through the region containing the simulated lymph nodes and static images of these are shown in Fig. 3.

Image display was done for 3-D views in Paraview (Kitware Inc.), using the processed phosphorescence image stack and the MIP images from the final two sets of tube experiments. Specifically, the experiments conducted with simulated skin were used for the 3-D reconstructions. The images were registered using the centroids of the Eppendorf tubes and edge of the phantom as fiducials to coalign the co-ordinate system of the CT to that of the phosphorescence images.

## 3 Results

**Fig. 2:**
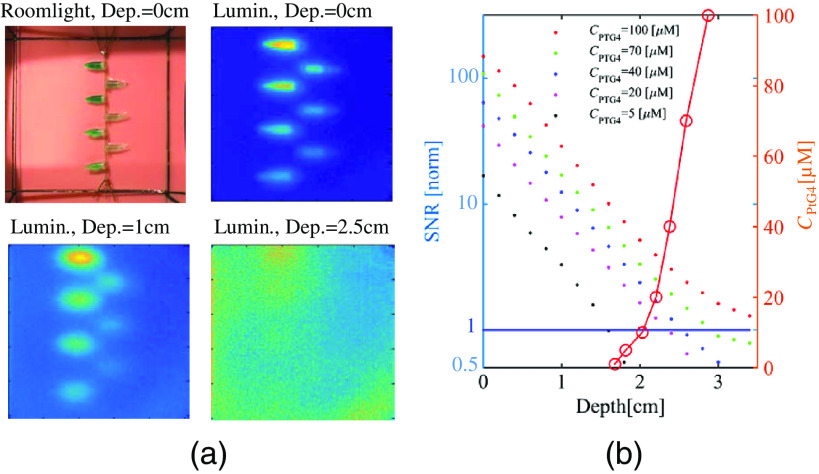
Experimental results to investigate image quality versus depth for different concentrations of PtG4 ($C_{\mathrm{PtG}4}$) as shown in the legend, showing (a) the room light image and luminescence images of the phantom used at three different depths in the phantom, and (b) the corresponding SNR values extracted from these images are shown as a function of depth by colored dots. In the same graph, the red line shows the $C_{\mathrm{PtG}4}$ at which SNR=1 (values on left y-axis).

In the depth and concentration ranging studies, CELSI experiments were performed for an intralipid-blood phantom embedded with PtG4-containing 0.5-mm Eppendorf tubes. The results are shown in Fig. 2, where sample luminescence and roomlight images are included in (a), and SNR is plotted in (b). The SNR has an exponential shape and the values plateau to their lowest values at deeper depths, as anticipated, because at some extreme depths the presence of the luminescent region cannot be sensed. Consequently, the SNR should be expected to asymptote to 1.0 at deep depths. The relationship between imaging depth and concentration of PtG4 for SNR=1 is described by the circled plots in Fig. 2(b), indicating that for depths <1.7  cm there was good SNR, but below this depth, the concentration required for SNR>1 increased dramatically. However, signal as deep at 3 cm was detectable, at concentrations just above 100 microMolar.

For the first set of geometry studies investigating phosphorescence of tubes containing PtG4 relative to control tubes containing PBS and a blank, a strong phosphorescent signal was detected. This was anticipated because the stock solution of 25  μM was a high concentration and was placed at a depth of only 3 mm. At 144 AoC, the SNR was computed to be 59, whereas at 60 AoC the SNR was 66. Overall, the 144 and 60 AoC cases produced similar results, with very similar SNRs, for the experiments with “skin.”

For the second set of experiments investigating different concentrations of PtG4, visible phosphorescent signals were detected from the 5- to 25-μM tubes, whereas the 1-μM tube did not produce a signal with sufficient SNR to be detected. The trend in signal strength, with respect to concentrations on the MIP images was as anticipated, with greater concentrations of PtG4 yielding stronger, brighter signals, with slight variation largely due to the thickness of skin overlying the tubes. Videos of the two sets of geometry studies are illustrated.

**Fig. 3:**
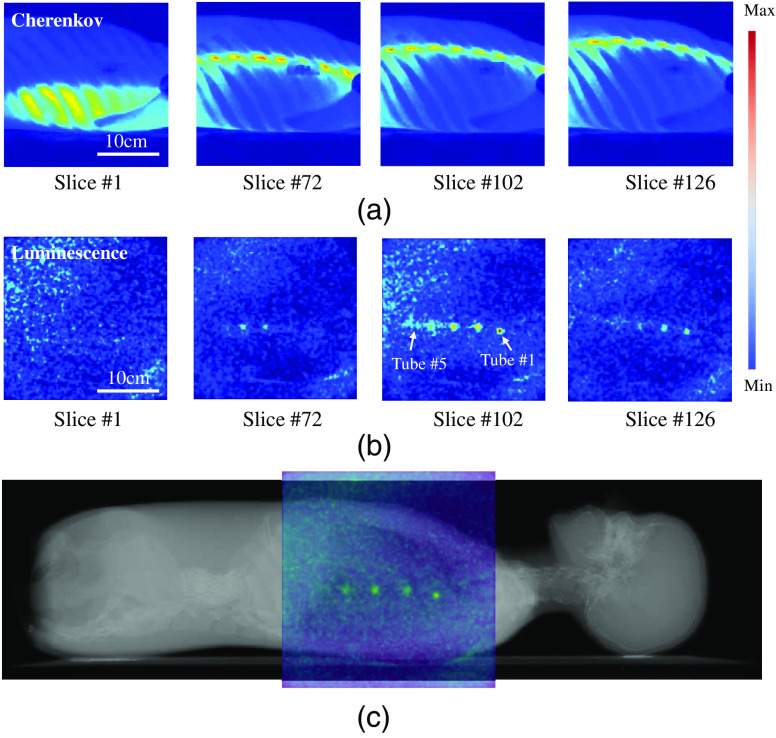
Raw measurement sequences are shown for (a) Cherenkov emission and (b) luminescence photons, for the sheet at four different slice locations. In (c) a composition image is shown of the phantom CT with color overlay of the CELSI MIP image. A temporal sweep of the signals in (a) and (b) can be seen in the associated video files as the sheet moved across the phantom body.

**Fig. 4:**
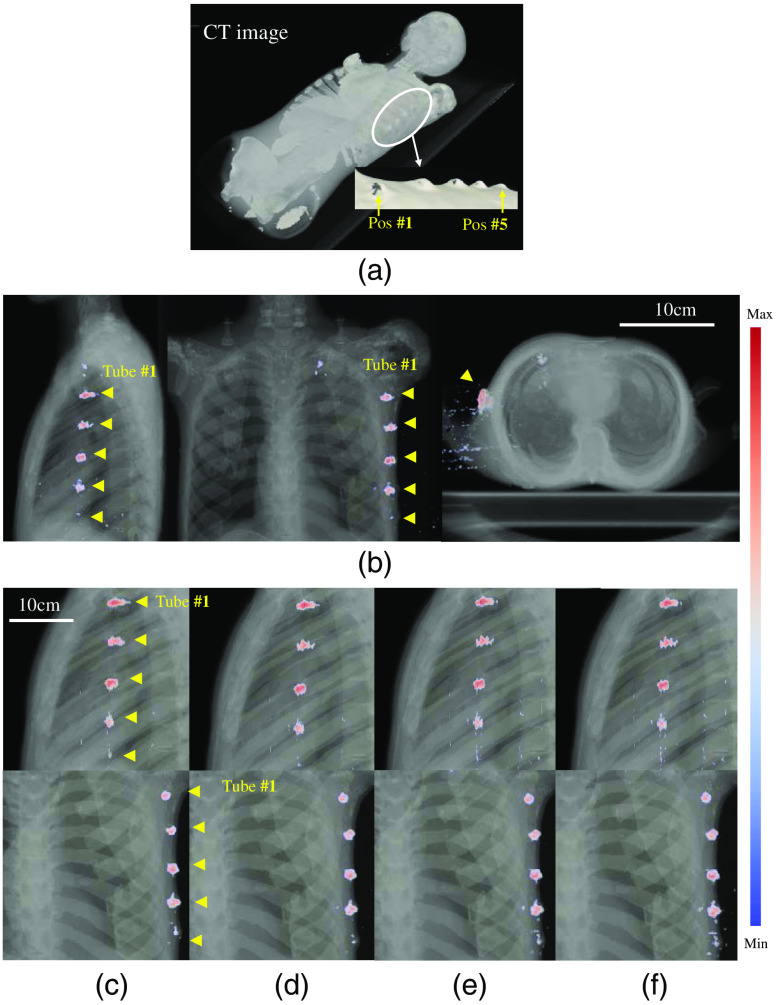
3-D images of (a) CT and (b) overlaid with CELSI for three orthogonal views: coronal, sagittal, and transverse. Compositions of CT and CELSI images for varying numbers of light sheets, which correspond to less dose delivery. In these images, the number of sheets were: (c) 25, (d) 50, (e) 100, and (f) 250, respectively.

The angular sheet-beam scanning methodology that is more suitable for imaging a chest region was used, as shown in Fig. 1. Raw measurements of Cherenkov light and luminescence for four specific slice positions are shown in Figs. 3(a) and 3(b), respectively. Figure 3(c) shows a combined image of CT and luminescence maximum intensity projection (MIP). Scanned image data were reprocessed and overlaid on the 3-D volume of the CT scan, to allow visualization of the recovered positions of the tubes. The recovered images are shown in Fig. 4, with a perspective view in (a), and the three orthogonal views of the CT in (b).

To pursue a minimum radiation dose, sparse illumination was investigated, i.e., the number of light sheets was desired to be as few as possible. Here, a total of 25, 50, 100, and 250 light sheets were used with equal spacing, i.e., with spaces 2, 1, 0.5, and 0.2 mm, respectively. Figures 4(c)–4(f) show the compositions of CT and CELSI images for different numbers of light sheet. It can be seen that the image quality was reasonable, even for 25 light sheets.

## 4 Discussion

Initial studies of depth sensitivity have indicated that CELSI imaging would be viable for a depth of perhaps 2 to 3 cm, likely dependent upon the tissue type involved. Adipose tissues tend to have lower blood volume and consequently reduced absorption attenuation of the light—this may allow imaging down to 3 cm. In contrast, denser tissues would have higher absorption and therefore be more limited to >2  cm.

The set of experiments conducted here were initially focused on determining the optimum positioning of the body phantom, couch, gantry, and camera for maximal sensitivity. Three orientations were investigated such as (i) vertical with the gantry directly above the body phantom on the couch at 180-deg reference angle and the camera placed directly facing the left side of the body phantom, (ii) horizontal with the gantry directly facing the left side of the body phantom at 90-deg reference angle and a 45-deg front-surface mirror placed above the top surface of the body phantom, allowing horizontal mounting of the ICCD directly facing the mirror, and (iii) angled with the gantry at 145-deg reference angle with ICCD on the same side. The angular orientation in an epi-illumination configuration was determined to be optimum and used for all subsequent experiments. In this orientation, a Cherenkov sheet was clearly visible translating through the body phantom. This orientation was also hypothesized to result in a strong phosphorescence signal from the simulated lymph nodes, and better approximated tangential breast tumor treatment protocol. The recovery of intensity was highly modulated by the depth of tissue overlying the tubes, and it was apparent that the signal was not linear with concentration because of this effect. However with skin thickness measured by the CT scan and geometry, it is feasible to quantify these values based on depth-dependent attenuation correction.^10^

These studies illustrate how imaging of lymph node sized objects may have potential with CELSI imaging in radiotherapy. Sensing their oxygenation is feasible, and further exploration of agents and signal analysis could lead to the next clinical trials in molecular sensing in radiotherapy.
